# How academic performance influences social integration: The moderation effect of cultural distance among Chinese cross‐borderers

**DOI:** 10.1002/brb3.2759

**Published:** 2022-09-14

**Authors:** Hu Jieyi, Cheung Chau Kiu, Xie Baojian

**Affiliations:** ^1^ School of Humanities Jinan University Zhuhai Guangdong China; ^2^ Department of Social and Behavioral Sciences City University of Hong Kong Hong Kong China; ^3^ School of Economics Jinan University Guangzhou Guangdong China

**Keywords:** academic performance, perceived cultural distance, social integration

## Abstract

**Background:**

Internal migration or cross‐border migration differs from traditional migration. The influence of academic performance on social integration among migration or cross‐border student groups has drawn attention.

**Method:**

A survey collected data from cross‐border students in Mainland China.

The sample included 616 university students (bachelor's, master's, and doctoral students) coming from Hong Kong studying in Guangzhou, Guangdong Province.

**Results:**

The moderating effect of cultural distance in the relationship between academic performance and social integration was significantly negative (*β* = –0.081, *p* *< 0.05*). The effect of academic performance on social integration was significantly positive (*β* = .104, *p* < .05). Length of time studying in the Mainland, social status, entrance exam score (which might affect the current academic performance), and acquiescence are as the control variable in examining the role of cultural distance in the effect of academic performance on social integration. This result embodies the functionalist theory.

**Conclusion:**

The host society is the structural whole requiring the function of social integration, whereas education is the structural component fulfilling the function. When cultural distance is large, the function of education for social integration decreases. The practical implication for enhancing social integration is relieving or bridging the distance.

## Graphical abstract

1. This study found that perceived cultural distance negatively moderated the contribution of academic performance to social integration.
2. Perceived cultural distance as a psychological factor may influence the contribution of social integration, which is the organizational support to adjustment.
3. The significance and contribution of this study lie in its demonstration of the usefulness of functionalist theory in explaining the moderation effect of perceived cultural distance on the contribution of academic performance to social integration.

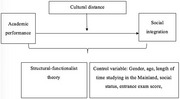

## 1 INTRODUCTION

After the reunification of Hong Kong with China and the development of the Guangdong–Hong Kong–Macao Greater Bay Area, the interaction between Hong Kong and Mainland China has surged (Lam, 2017). A prominent phenomenon is the increasing number of Hong Kong youth who cross the north border to the Mainland to attend universities (Li, 2011). However, after coming to the Mainland, these students from Hong Kong face difficulties in social integration because of the cultural distance, language barrier, and differences in customs and values (Na and Hample, 2016). The significance and contribution of this study lie in its demonstration of the usefulness of functionalist theory in explaining the moderation effect of perceived cultural distance on the contribution of academic performance to social integration.

Internal migration or cross‐border migration differs from traditional migration (Guo et al., 2018). Migration, which is movement for resettlement, is one of the most complex components of demographic change (Bell et al., 2002). Internal migration has become more popular than international migration in most countries (Guo et al., 2018). This study fills the research gap about social integration among cross‐borderers. Such integration is noteworthy in cross‐border migrants between Hong Kong and the Mainland (Li, 2011). The similarity between internal migration and traditional migration is migrants pursuing more life chances. Cross‐borderers are not yet immigrants because they may not care about their permanent resident status. Moreover, some of them are more likely to go back to their hometown in the recent future.

Social integration is a significant concept in migration research, and scholars always relate social integration to other factors to analyze predictors of social integration. Social integration refers to relationships or ties binding people together to uphold social belonging and inclusion (Wray et al., 2011). Social integration is not only a situation where minority groups come together or integrate into mainstream society, but also a process of agreement on a shared system of aspects such as meaning, language, and culture (Echenique & Fryer, 2007; Forrest & Kearns, 2001). Integration evolves from social growth, structural development, functional differentiation, and interdependence (Spencer, 1947). Notably, social integration indicates interdependence between the various parts of social structure and the coordination and control of these parts (Spencer, 1947).

Diminishing cultural distance is also the psychological process of social integration (Yue et al., 2016). Identities, cultural distance (norms, values, cultures, customs, and differing views), public awareness, ethical sensitivity, and motivation all influence migrants’ process of social integration after migration (Sheu & Fukuyama, 2007). Interaction between Hong Kong and Mainland China is increasingly frequent after reunification. Cross‐border students, who come from Hong Kong to attend universities in the Mainland, face difficulties in integration because of differences in language, custom, and habit, while their social integration in the Mainland buttresses their well‐being and health (Na & Hample, 2016). The definition of cultural distance, given by Triandis (1994), concerns differences in mother tongue, religion, family and marital life, and values between cultures. Perceived cultural distance presents difficulty in the social integration process, thereby impedes social integration. However, the role of perceived cultural distance in the relationship between academic performance and social integration remains unknown.

Functionalist theory frames the relationship between academic performance and social integration moderated by perceived cultural distance. The components of society constitute the structural whole (Cheung and Leung, 2009). Thus, education is the structural component and social integration representing the whole (Cheung and Leung, 2009). Given that the criteria of higher academic performance might not be similar in the different cultures, cultural distance may influence the evaluation of academic performance between the host society and hometown from the perspective of cross‐borderers. Cultural distance may influence the relationship between academic performance and social integration.

## 2 LITERATURE REVIEW

The current study addresses existing gaps in the literature. First, the measurement of social integration remains uncertain in the extant research. The measurement of social integration initiated by Park and Burges (1970) had the four dimensions of economic competition, political conflict, cultural integration, and social connection, while Landercker (1951) classified these measurements into cultural, functional, normative, and communicative integration. The common dimensions of social integration are identity (Chen et al., 2015; Ren & Qiao, 2010; Yang, 2010), economic (Yang, 2010), cultural (Yue et al., 2016, pp.79), psychological (Yang & Qin, 2016), and community integration (Lou & He, 2009).

Second, a gap exists regarding the linkage between academic performance and social integration. The moderators or mediators in the effect of academic performance on social integration still need further examination. Education is a structural part of the society, sustaining social integration specifically in a relevant place. Education is measured by educational outcomes, particularly presented by academic performance. Social integration is also a crucial research focus for cross‐border groups. Therefore, the type of students that can integrate better is a research question that needs an answer for providing implications for government to formulate the “talent absorbing” policy (Shao et al., 2022). This study chose one characteristic that is a distinctive feature of students, namely, academic performance, to examine the relationship with social integration.

Finally, perceived cultural distance may exert effects on social integration and the relationship between academic performance and social integration. In these relationships, academic performance is the social factor of social integration, whereas perceived cultural distance is the psychological factor of social integration. According to the structural–functionalist theory, education as a structural component of the social system needs to be culturally relevant because the standard of educational success varies across cultures. Consequently, the relevance of education to another culture diminishes with cultural distance.

### 2.1 Social integration

The term “integration” was first introduced in the writings of biological evolutionists. Blau (1994) argued that the achievement of social integration is a key issue in modern society. The main factor of social integration is attraction, which is not about appearance but rather how well people deliver their own value to a community. The first step of seeking a career, rather than just a job in the Mainland, will be social integration in the Mainland. Social integration helps one to achieve successful careers more easily. One dimension of social integration is cultural integration, which can relate to Berry's conceptualization of acculturation expectations (Berry, 2001). The conceptualization categorizes acculturation strategies along these two dimensions to provide assimilation, separation, integration, and marginalization (Berry, 1997). Assimilation and integration mean adopting the culture including language and other lifestyles of the host society (Rudmin, 2003). More than such cultural integration, social integration incorporates fitting and cooperation with host society even without cultural integration ( Arends‐Tóth & van de Vijver, 2006; Chataway & Berry, 1989; Ward, 2001; Ward & Geeraert, 2016).

Social integration depends on demographic factors (age, gender) (Munck, 2009), social factors (Cattan & Ingold, 2003), and psychological factors (Nicholson et al., 2014). Specifically, the demographic predictors of social integration include age, gender, educational level, marital status, and religious faith. Social predictors include adjustment skills, academic performance in students’ groups, and social network. Cattan & Ingold (2003) related social networking to social integration among older persons in the community. Social networking influences social integration in that relationships with locals present the situation of social integration. In addition, social support (Beckley, 2006; Bloom & Spiegel, 1984; Friedland & McColl, 1987; Kelly‐Hayes & Paige, 1995) and cognitive appraisals such as self‐efficacy (Cunningham et al., 1991; Schiaffmo & Revenson, 1992) also influence social integration by attaining material and social resources. Depressive symptoms, religious engagement, and perceived cultural distance represent psychological factors in affecting social integration (Nicholson et al., 2014).

Academic performance is a distinctive feature of students and a supposed determinant of social integration. Tinto (1997) identified academic performance as a crucial link between interaction or engagement and learning outcomes. From a broader view, academic performance and social integration are integral to cross‐border students’ well‐being. The contribution of performance to the integration therefore merits investigation.

### 2.2 Effects of academic performance on social integration

One of the supposed functions of education is enhancing the social integration or identification among students, especially immigrant students (Hwang, 2013). Particularly, education counts as a structural part of society from the perspective of the structural–functionalist theory (Shaidullina et al., 2015). This theory holds that education is functional to the maintenance of social solidarity. Effective education contributes to the student's belongingness to present an integrative function to society (Rest et al., 1999).

Cross‐borderers taking educational classes, such as language classes, literacy classes, and employment training programs, are more likely to integrate into the local culture and society (Chen et al., 2015). Some scholars defined the act of “interaction with faculty or peers” as integration among students, which is a type of academic achievement (Tinto, 1993). Others even regarded it as social integration (Braxton et al., 2000). Tinto (1975) used the term academic performance to describe the ability of students’ academic achievement related to institutional expectation. However, Tinto (1997) and Tinto et al. (1994) demonstrated that integration in class among migrant student groups greatly fosters social integration.

University factors aim to achieve educational outcomes (Buchmann & Hannum, 2001). University is a multilevel learning environment, contributing to social sustainability through inserting values in education, especially influencing students’ attitudes after graduation (Sherman & Hansen, 2010; Tilbury, 2011). For example, academic institutions fulfill the need to construct a framework for educating students to contribute to sustainable development with the value of sustainability (Godemann et al., 2011), which originates from faculty support for sustainable integration (Maloni et al., 2012; Walck, 2009). Many Hong Kong young adults attend universities in the Mainland to shape their social integration in the future, building on their social integration initiated in the universities (Shao et al., 2022). As such, universities tend to incorporate the values of social responsibility and sustainability in education. Thus, the students after graduation are more willing to integrate into the local environment rather than to subvert it (Chen et al., 2015). Universities help students understand others’ values and culture and promote awareness about human rights and diversity to let them embrace other cultures more easily (Engel et al., 2013).

Educational outcomes typically include attainment and achievement (Cuesta et al., 2016; Glewwe et al., 2011; Mitchell et al., 2008; Snilstveit et al., 2015). According to the situation of universities in the Mainland, educational achievement is more associated with social integration as enrolment rates are always stable with Hong Kong migrants coming to the Mainland (Hu and Cheung, 2021), whereas attainment is greatly susceptible to family education. In terms of the educational achievement, the major measurement is students’ academic performance (Hu and Cheung, 2021).

### 2.3 Moderation effect of perceived cultural distance on the contribution of academic performance to social integration

Perceived cultural distance is likely to affect social integration. Perceived cultural distance is threatening and leads to “reactive distinctiveness,” a form of prejudice against immigrants (Jetten et al., 2004).

Cultural distance between social groups has been counted as a crucial predictor for intergroup attitudes (Allport, 1954), resulting in discrimination and social exclusion (González et al., 2008). Intergroup attitudes obviously influence relationships or ties binding people together, which is the definition of social integration proposed by Wray et al. (2011). Furthermore, Guan et al. (2011) and Lam et al. (2006) found that a larger perceived cultural distance has been associated with stronger feelings of threat and more negative attitudes toward the outgroup. However, perceived cultural difference not only directly connects with feelings of threat but can also moderate the effect of multiculturalism (Mahfud et al., 2018). The multicultural goal is promoting tolerance for other cultures. Nevertheless, interestingly, recognizing and accepting cultural differences are easier given a larger perceived cultural distance (Mahfud et al., 2018).

Perceived cultural distance as a psychological factor affecting social integration is likely to influence the contribution of academic performance to social integration. In this review of expatriate literature, Thomas (1998) also conducted research on how cultural distance may influence the contribution of organizational support to adjustment. Meta‐analyses have found that perceived cultural novelty or distance in studies of expatriates and international students impairs social integration (Black & Mendenhall, 1991; Stahl & Caligiuri, 2005; Wilson et al., 2013). Suanet and Van de Vijver (2009) and Galchenko and van de Vijver (2007) examined the role of perceived cultural distance in the acculturation of exchange students in Russia, whereas Babiker et al. (1980) explored the relationship between cultural distance and performance in overseas students at Edinburgh University.

The moderation effect of perceived cultural distance is viable according to the functionalist theory. Mahfud et al. (2018) noted that perceived cultural distance affected attitudes toward immigrants. Therefore, cultural distance influences the process of social integration because of the diverse standards. The findings of Adetimirin and Omogbhe (2011) indicated that the use of library services among distant‐learning students, who did not interact with peers, moderated their performance in the class and their class integration. People tend to be motivated to perceive their intergroup as distinctive from other groups (Tajfel and Turner, 1979), which affects other factors influencing integration (Guan et al., 2011).

## 3 THEORETICAL FRAMEWORK: FUNCTIONALIST THEORY

The effects of cultural distance on the relationship between academic performance and social integration are justifiable by the structural–functionalist theory. Generally, structural–functionalist theory indicates that the sustenance of a social system requires the integrative functioning of structural components (Ford & Lerner, 1992). The functioning of society depends on the components in the structural whole with positive feedback (Cheung & Leung, 2009). In this case, the host society is the structural whole requiring the function of social integration, and education is the structural component fulfilling the function. Education, particularly indicated by academic achievement, is functional to society, thereby sustaining social integration specifically in a relevant place and treasuring education and achievement (Snilstveit et al., 2015). Cultural distance comprises differences in language, custom, lifestyle, and values between the host and origin societies (Taušová et al., 2019). The theory suggests that when the cultural distance is larger, the function of education in social integration is smaller (Galchenko & van de Vijver, 2007; Wilson et al., 2013). This moderation occurs because the host society does not appreciate education or achievement attained in the origin society (Mitchell et al., 2008). Conversely, if cultural distance is shorter, academic performance can have a stronger contribution to the host society and social integration there.

**FIGURE 1:**
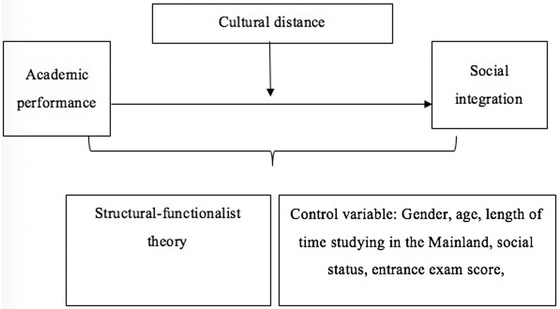
The theoretical framework of the relationship between academic performance and social integration

The theoretical framework shows as follows. (Figure 1). Given the above literature review of the relationship between academic performance and social integration and the theoretical framework, the related hypotheses are:

H1 Academic performance positively associates with social integration.

H2 Perceived cultural distance negatively moderates the relationship between academic performance and social integration.

## 4 METHOD

A survey collected data from cross‐border students in Mainland China.

### 4.1 Participants

The sample included university students (bachelor's, master's, and doctoral students) coming from Hong Kong studying in Guangzhou, Guangdong Province. Guangdong is adjacent to Hong Kong and they share a similar culture, including customs, accent, and values. The survey in Chinese, distributed to diverse institutes and their departments and classes, applied to students coming from Hong Kong only. The sample consisted of 616 students from Hong Kong. Among them, 40.3% were male students, and 59.7% were female students. The majority age group was that from 18 to 25 years (*N* = 587). In addition, the time, studying in the Mainland China as the control variable, used months as units to measure the time they cross‐border until the month of participating in the survey. The average length of time staying in the host society was 99.54 months.

### 4.2 Measurement

#### 4.2.1 Social integration

A number of social integration scales (Ellison et al., 2007; Ross et al., 2009) were usable and revised to apply in this research. A total of 19 items regarding social integration (with 5 reversed items) had two dimensions, “with natives” and “with community.” Example items include “You shared information with natives,” “You discussed about social life with natives,” and “You talked about integration into the Mainland with natives” in the most recent month (Table 2). The survey used the timeframe in the questionnaires to avoid the endogeneity problem in the relationships between the two major variables: academic performance and social integration. The grade‐point average (GPA) part of academic performance is last school year's GPA, whereas social integration asked about feelings in the most recent month.

#### 4.2.2 Academic performance

This study measured academic performance with the student's GPA and awards. Specifically, they were the GPA and the prizes (national level, city level, and school level) received in the last semester. The sum of their standard scores then measured academic performance.

Notably, academic performance was that of the last semester, whereas social integration referred to that of the recent month. Therefore, academic performance occurred before social integration in this study.

#### 4.2.3 Perceived cultural distance

Based on Muthukrishna et al.’s (2020) study, each student rated the perceived difference between the host (Mainland China) and origin society (Hong Kong) on a 0−10 scale in the last year.

#### 4.2.4 Acquiescence

Acquiescence is a measure required for controlling for response bias in rating (Baumgartner & Steenkamp, 2006; He et al., 2017). Without the controlling, the bias could inflate relationships among ratings (Clarke, 2001; Dolnicar & Grun, 2007). At worst, acquiescence could account for one‐quarter of the total variance in a rating (Baumgartner & Steenkamp, 2006). Bachman & O'Malley (1984) suggested the average of ratings as a measure of acquiescence. Controlling of this has been useful to reduce ambiguity in findings based on ratings (He & Van de Vijver, 2016).

#### 4.2.5 Academic performance at the time of admission

Academic performance for university admission could be that obtained from Gaokao, Danzhao, or Joint examination. It was transformable to a 0−100 scale.

#### 4.2.6 Length of time studying in Mainland China

Although all the participants were university Hong Kong students studying in Mainland China, the time when they started studying in Mainland China varied thereby potentially affected social integration. Though all the participants are university Hong Kong students studying in Mainland China at the time doing the questionnaire, the time of starting studies in the Mainland China is different, which might affect social integration. Herein, the length of time of studying in Mainland China should be used as a control variable in the model.

### 4.3 Procedure

Statistical product and service solutions (SPSS 25.0) was used to analyze all the data. Each potential participant was found by tutors from different universities or by convenience sampling in Guangdong Province, Mainland China. Hierarchical regression analysis was applied to hypothesis testing. The problem of multicollinearity was negligible, considering high tolerance (> 0.3). Specifically, it tested the effects of academic performance on social integration and its moderation effect by cultural distance. The consent form included the information regarding the objectives of the study, information about the researcher, and so on. All the participants were informed of the purpose of this study and the process of the survey. They were then given the power to decide whether they wanted to participate. Moreover, the participants can stop the survey whenever they felt uncomfortable. All the answers in the questionnaires remained confidential. Ethical approval has been obtained before the survey.

## 5 RESULTS

The means (SDs) of academic performance, social integration, and cultural distance were 5.13(1.353), 3.35(0.635), and 6.25(1.941) respectively. Social status is the question asking the socioeconomic class in terms of household income from 0(low) to 10(high). Social integration was the average of five rating items, scored from 0 to 100. Particularly, the items of social integration had 0 for “very little,” 25 for “little,” 50 for “average,” 75 for “rather a lot,” and 100 for “very much.” (Table 1)

**TABLE 1:** Personal characteristics (*N* = 616)

	Coding	*N* (%)	*M* (SD)
**Gender**			
Male	0,1	248 (40.3)	
Female	0,1	368 (59.7)	
**Age**			
Under 18	0,1	16 (2.6)	
18–25	0,1	587 (95.3)	
26–34	0,1	7 (1.1)	
Over 35	0,1	6 (1.0)	
**Length of time studying in the Mainland**	Months		99.54 (84.498)
**Social status**	0–10		4.39 (1.879)
**Academic performance**	0–4		2.13 (1.353)
**Social integration**	0–100		3.35 (0.635)
**Cultural distance**	0–10		6.25 (1.941)

The distributions of all the variables did not deviate considerably from the normal distribution (according to their skewness and kurtoses). Their composites also presented acceptable reliability (*α* > .70). All the variables were thus suitable for regression analysis. (Tables 2, 3, 4, 5)

**TABLE 2:** Mean, standard deviation, skewness, and kurtosis of the items of social integration

Item	M	SD	Skewness	Kurtosis
You shared information with natives	3.34	1.156	–0.279	–0.625
You discussed about social life with natives	3.37	1.150	–0.294	–0.650
You discussed about career goals with natives	3.04	1.179	–0.016	–0.804
You talked about integration into the Mainland with natives	2.97	1.243	0.075	–0.905
You and natives were close to each other	3.45	1.066	–0.313	–0.417
You joined recreational activities together with natives	3.42	1.162	–0.318	–0.677
NOT‐You and natives kept innermost thoughts to themselves	3.15	1.081	–0.129	–0.473
NOT‐You and natives were separate groups	3.30	1.155	–0.125	–0.751
You and natives solve problems together	3.33	1.038	–0.179	–0.460
NOT‐You and natives avoided each other	3.72	1.108	–0.614	–0.272
You and natives had affection to each other	3.41	1.073	–0.248	–0.432
You used school services easily	3.43	.948	–0.247	–0.126
You obtained basic resources from the local community easily	3.20	.991	–0.097	–0.176
The local community was a source of comfort	3.29	.963	–0.144	.007
NOT‐You felt strange to the local community	3.65	1.092	–0.497	–0.378
You were familiar with local customs	3.48	0.978	–0.145	–0.406
You and natives shared the same values	3.26	0.971	–0.123	–0.073
NOT‐You hated following local rules	3.69	1.121	–0.528	–0.475
The local resource is basically fair to you and natives	3.18	0.936	–0.087	0.160
Cronbach's Alpha = 0.885				

**TABLE 3:** Factor analysis for social integration

Item	Factor loading	M	SD
You discussed about social life with natives	0.881	3.37	1.150
You shared information with natives	0.817	3.34	1.156
You discussed about career goals with natives	0.805	3.04	1.179
You and natives were close to each other	0.803	3.45	1.066
You joined recreational activities together with natives	0.803	3.42	1.162
You and natives solve problems together	0.746	3.33	1.038
You and natives had affection to each other	0.710	3.41	1.073
You talked about integration into the Mainland with natives	0.699	2.97	1.243
The local community was a source of comfort	0.795	3.29	.963
You obtained basic resources from the local community easily	0.773	3.20	.991
The local resource is basically fair to you and natives	0.711	3.18	.936
You used university services easily	0.672	3.43	.948
You and natives shared the same values	0.648	3.26	.971
You were familiar with local customs	0.560	3.48	.978
NOT‐You and natives avoided each other	0.798	3.72	1.108
NOT‐You and natives were separate groups	0.784	3.30	1.155
NOT‐You felt strange to the local community	0.772	3.65	1.092
NOT‐You and natives kept innermost thoughts to themselves	0.733	3.15	1.081
NOT‐You hated following local rules	0.692	3.69	1.121

**TABLE 4:** The second‐order model of factor analysis for social integration

Components	Factor loading	M	SD
With natives	0.912	3.29	.946
With community	0.912	3.31	.735

*Note*: “With natives” included the items: You discussed about social life with natives; You shared information with natives; You and natives were close to each other; You discussed about career goals with natives; You joined recreational activities together with natives; You and natives solve problems together; You and natives had affection to each other; You talked about integration into the Mainland with natives, etc. “With community” included the items: The local community was a source of comfort; You obtained basic resources from the local community easily; The local resource is basically fair to you and natives; You used university services easily; You and natives shared the same values; You were familiar with local customs. The order of items was determined by the factor loading size with higher factor loading front, etc.

**TABLE 5:** Regression analysis of social integration

	Standardized coefficients		Tolerance	
Independent variable	Model 1	Model 2	Model 1	Model 2
Academic performance	0.102*	0.116**	0.884	0.856
Female	–0.075	–0.080*	0.893	0.983
Age	0.021	0.017	0.848	0.967
Length of time studying in the Mainland	0.233***	0.227***	0.918	0.914
Social status	0.102*	0.106**	0.819	0.873
Cultural distance	–0.057**	–0.055**	0.927	0.948
Entrance exam score	–0.045	–0.044	0.929	0.920
Acquiescence	0.169***	0.176***	0.860	0.858
Academic performance × Cultural distance		–0.082*		0.923
R^2^	0.136	0.153		

* *p* < 0.05.

** *p* < 0.01.

*** *p* < 0.001.

The moderating effect of cultural distance on the effect of academic performance on social integration was significantly negative (*β* = –0.081, *p* < 0.05). The effect of academic performance on social integration was significantly positive (*β* = 0.104, *p* < 0.05). In addition, social status had a significant positive influence on social integration (*β* = 0.104, 0.106, *p* < 0.05, .01). Acquiescence (*β* = 0.167, 0.173, *p* < 0.001) presented a significant positive effect on social integration in both model 1 and model 2. Length of time studying in the Mainland, social status, entrance exam score (which might affect the present academic performance), and acquiescence are as the control variables in examining the role of cultural distance in the effect of academic performance on social integration. All the tolerances were acceptable in the regression models. The regression models explained 13.7% and 14.3% of variance in social integration.

Consequently, academic performance positively influenced social integration and perceived cultural distance had a negative impact on social integration. This result also supported H2 that perceived cultural distance negatively moderated the contribution of academic performance to social integration.

## 6 DISCUSSION AND IMPLICATION

Perceived cultural distance is likely to influence social integration. Several dominant cultural values in Asians include collectivism, emotional self‐control, family recognition, conformity to norms, and humility (Yang, 1999). These values were also the focus of the Asian migrant groups. For this reason, the present study fills the research gap to explore the perceived cultural distance in Asian cross‐borderers. In other words, perceived cultural distance between Hong Kong and the Mainland is a predictor of low social integration. Cross‐border students from Hong Kong studying in Mainland universities showed better social integration with better prior academic performance, especially when perceived cultural distance was smaller. The larger the cultural distance, the greater the difficulties for the student to enjoy social integration and reap its benefits in academic performance.

The difference between Hong Kong and the Mainland, or the perceived cultural distance between the students’ hometown and the study place, negatively moderated the contribution of academic performance to social integration. Findings showed that the larger the cultural distance, the greater the difficulty for individuals to achieve social integration. The important moderating effect of perceived cultural distance on the function of academic performance for students’ social integration is consistent with past research findings. For example, the role of perceived cultural distance had a strong linkage with personal growth initiative and language proficiencies among the international student group (Taušová et al., 2019). Specifically, perceived difference between the host and origin societies hinders the contribution of academic performance to social integration. With high perceived cultural distance, the function of education in social integration decreases.

The findings provide additional support to existing literature that education can play a crucial role in promoting social integration (Putnam, 2000). Education is strongly conducive to a variety of social outcomes, such as social engagement and social integration. Moreover, education, as the effective component in a host society, has the function of social solidarity, namely, social integration (Shaidullina et al., 2015). Moreover, Roberts‐Schweitzer et al. (2006) also suggest that education empowers individuals to increase their knowledge and cognitive and social skills, as well as improve their values and attitudes toward social integration. Education helps students make informed decisions competently by improving their socio‐emotional capabilities (e.g., social and emotional skills). As such, education helps individuals increase their interest in social engagement and even social integration and understand benefits brought by migration.

These findings support the structural–functionalist theory about the function of education to host society. This result aligns with that about the contribution of academic performance to social integration (Tinto, 1997). This study further explores the moderation of perceived cultural distance to the contribution. Cultural distance may depreciate the value and thus the function of academic performance to host society (Mahfud et al., 2018). The structural–functionalist theory explains the contribution of academic performance to social integration with the vision on the functions of structural parts to uphold the whole (Cheung and Leung, 2009). This study embodies the structural–functionalist theory, which suggests academic performance is a component of the social system to buttress social integration (Hwang, 2013; Shaidullina et al., 2015). Cultural distance may influence the standard of academic performance or even other successes (Suanet and Van de Vijver, 2009). In other words, people in host societies discount education or achievement attained from origin society because of cultural distance. Therefore, education is a structural part of the social whole, and the educational function varies with cultural distance.

There are several implications that need to be addressed. First of all, this study can extend the understanding of social integration in Asian groups, Hong Kong young adults. Moreover, this study can also enrich the knowledge of aspects such as academic achievement, adaptability, and social engagement, especially in terms of their contributions to social integration. In addition, the findings may be particularly important to policymaking for cross‐border students. Perceived cultural distance dampens social integration and its response to academic achievement among cross‐border students from Hong Kong. These findings generate practical implications for practitioners concerned with social integration, such as policymakers and educators. Accordingly, educators can set up criteria for success, mainly students’ academic performance, as defined by society (Roberts‐Schweitzer et al., 2006). Government or policymakers should establish a talent‐rating system, attracting students with a small cultural distance from the host society. Universities should also arrange activities for cross‐border students to shorten perceived cultural distance. Migration or cross‐border students also need to take the initiative to integrate into the host society by diminishing cultural distance from the society (e.g., accepting the culture in the host society). Furthermore, education cannot play a role in isolation because students only spend half of their non‐sleeping time in university. Policymakers easily underestimate the function of home and community environments. For example, if the community cannot provide resources and opportunities for social integration sufficiently, students do not have sufficient space and chances for social integration, even though they want to integrate into the host society.

## 7 LIMITATIONS AND FUTURE RESEARCH DIRECTION

First, all the participants studied in Guangzhou, Guangdong Province in China, which might be different from other provinces in China in dialect, custom, and other subcultural elements. Other provinces and even other countries can test the above model. The cultural distance experienced by Hong Kong Chinese students may be considerably smaller than that experienced by international students who are less familiar with the host context.

Second, the cross‐sectional design and self‐reported measures (particularly academic performance) of this study hinder the rigorous analysis of the relationship among academic performance, social integration, and perceived cultural distance. Given the limitations, panel research design is highly recommended in future research to examine causal relationships among these factors.

Last, the larger the cultural distance, the higher the difficulty for individuals to conceive belongingness. However, other cultural samples are necessary to clarify the effects of cultural factors, which further need qualitative data.

## CONFLICT OF INTEREST

The authors declare no conflict of interest.

## Data Availability

No
